# MicroRNAs and Triple Negative Breast Cancer

**DOI:** 10.3390/ijms141122202

**Published:** 2013-11-11

**Authors:** Elvira D’Ippolito, Marilena V. Iorio

**Affiliations:** Start Up Unit, Experimental Oncology Department, Fondazione IRCCS Istituto Nazionale Tumori, Via Amadeo 42, Milan 20133, Italy; E-Mail: elvira.dippolito@istitutotumori.mi.it

**Keywords:** microRNA, breast cancer, triple negative

## Abstract

Triple Negative Breast Cancer (TNBC) is a very aggressive tumor subtype, which still lacks specific markers for an effective targeted therapy. Despite the common feature of negativity for the three most relevant receptors (ER, PgR and HER2), TNBC is a very heterogeneous disease where different subgroups can be recognized, and both gene and microRNA profiling studies have recently been carried out to dissect the different molecular entities. Moreover, several microRNAs playing a crucial role in triple negative breast cancer biology have been identified, providing the experimental basis for a possible therapeutic application. Indeed, the causal involvement of microRNAs in breast cancer and the possible use of these small noncoding RNA molecules as biomarkers has been extensively studied with promising results. Their application as therapeutic tools might represent an innovative approach, especially for a tumor subgroup still lacking an efficient and specific therapy such as TNBC. In this review, we summarize our knowledge on the most important microRNAs described in TNBC.

## 1. Introduction

### 1.1. MicroRNA Biogenesis

MicroRNAs (miRNAs or miRs) are small non-coding endogenous RNA molecules involved in gene regulation, and are located at introns of protein-coding genes, introns of non-coding genes, or exons of non-coding genes [1,2].

In the canonical biogenesis pathway, microRNAs are transcribed by RNA polymerase II as a long primary transcript (pri-microRNA) and cleaved, in the nucleus, into a 60–70 nt double helix hairpin structure precursor (pre-microRNA) by the RNase III Drosha and its cofactor DGCR8 [3]. After Exportin 5-mediated transfer into the cytoplasm [4], the pre-microRNA is first processed by the RNase III Dicer, in concert with TRBP or PACT cofactors, in an approximately 22 nt dsRNA with two-nucleotide 3′-overhangs [5] called microRNA/microRNA*, and then unwound by cytoplasmic helicase. The strand with the lower stability in the 5′ end (guide strand) is preferentially selected and incorporated in the RISC (RNA-induced Silencing Complex), which includes Argonaute proteins, the microRNA strand and other additional factors, even if in some cases both strands could be incorporated. In this conformation the mature microRNA is directed toward target mRNA.

The binding can occur through a complete or partial base pairing, which respectively leads to degradation, mainly in plant, or translation inhibition, more frequent in mammals and often followed by deadenylation and subsequent degradation of the mRNA [6]. The binding for the most part occurs between the 5′-nucleotides 2–8 of microRNA, the so called “seed” region, and the 3′ untranslated region (3′UTR) of the mRNA, even if also the 5′UTR, promoter and open reading frame regions were demonstrated to be targetable [7,8]. Moreover, microRNAs, as well as dsRNAs, were found to be able to bind promoter regions at the genomic level and induce, rather than repress, gene expression [9,10] by returning in the nucleus, possibly by a hexanucleotide terminal motif mediated transfer [11].

The microRNA scenario is further complicated by the Drosha-independent [12] or Dicer-independent [13,14] mechanism of microRNA biogenesis, or by other kind of RNA molecules which can contribute to the microRNA pool; examples are the so-called mirtrons, RNA structures which resemble the pre-microRNA and do not need Drosha processing [15], or the small nucleolar RNA-derivered RNAs (snRNA) or the tRNA-derived RNAs (tdRNA), which have a totally independent biogenesis pathway [16].

There has been an interesting shift in the study of microRNA biogenesis from the simple elucidation of its key events to the utilization of its potential role as a pharmacological target. AC1MMYR2, a specific small molecule inhibitor of miR-21, for instance, has been designed exploiting the structure conformation of pre-miR-21; the molecule acts by specific blocking of Dicer-mediated miR-21 processing leading to tumor growth suppression and epithelial-mesenchymal transition (EMT) reversion, in epithelial tumor cells and orthotopic nude mouse model [17].

Genetic variants in genes of microRNA biogenesis pathways were associated with the risk and/or survival in various malignancies such as colorectal cancer [18], bladder cancer [19], renal cell carcinoma [20], ovarian cancer [21], head and neck cancer [22,23], prostate cancer [24] and breast cancer [25].

microRNAs can also be secreted in the surrounding region or secreted in biological fluids where they are protected in liposomal-like particles and act on other cell types. Indeed, an increasing body of evidence reveals how circulating miRNAs are associated to MVs (microvescicles), small exosomes/vesicles of endocytic origin released by normal healthy or damaged cell types. Valadi and colleagues demonstrated in 2007 how vesicles released from human and murine mast cell lines contain over 1200 mRNA and approximately 121 miRNA molecules [26], and a year later Taylor and colleagues [27] described a miRNA signature associated to tumor-derived exosomes. Further evidence of this was provided by several other studies.

Furthermore, the small size of miRNAs certainly contributes to a higher stability in comparison with mRNAs, allowing the study of their expression in fixed tissues or other biological material, and thus supporting their possible use as novel, minimally invasive and robust biomarkers: miRNAs can be reliably extracted and detected from frozen and paraffin-embedded tissues, from blood (either total blood, plasma or serum) [28,29], from circulating exosomes [30], and from different biologic fluids as urine [31], saliva [32] and even sputum [33].

### 1.2. MicroRNAs and Breast Cancer

After the first microRNA signature characteristic of breast carcinoma that our group [34] described in 2005, several of the most significant microRNAs differentially expressed have been extensively studied since their initial discovery and an important role on the biology of breast cancer was revealed. One of the first oncomiRs identified was miR-21, overexpressed in breast carcinoma, which mediates cell survival and proliferation, stimulates invasation, extravasation and metastasis [35] and which has been associated with advanced clinical stage, lymph node metastasis and patient poor prognosis [36,37]. Moreover, mice conditionally expressing miR-21 develop a pre-B malignant lymphoid-like phenotype, thus demonstrating that miR-21 is a genuine oncogene [38].

In contrast, down regulated microRNAs, such as miR-125a and b and miR-205, regulate oncogenes as tyrosine kinase receptors HER2 and HER3, respectively [39,40], whereas let-7 has been described as a new regulator of self-renewal and tumorigenicity of breast cancer cells [41], targeting molecules originally described in lung cancer: RAS, HMGA2 and MYC.

Notably, one of the first “metastamiR”, miRs involved in the metastatic process, has been described in a breast cancer model by Weinberg group, miR-10b [42], for which the same group has also explored a possible therapeutic application, reporting that systemic treatment of tumor-bearing mice with miR-10b antagomirs suppresses breast cancer metastasis [43]. Always focusing on metastasis-inducers microRNAs, the same group (Ma L. and colleagues) [44] described how miR-9 increases the metastatic potential, and miR-373 and miR-520c stimulate the non-metastatic MCF7 cell line to migrate and invade *in vitro* and *in vivo*, regulating the cell-surface glycoprotein CD44 [45].

Whereas, metastasis inhibitors are for example miR-126 and miR-335 [46] or miR-200 family members and miR-205, which have been shown to reduce cell migration and invasiveness targeting ZEB transcription factors, known inducers of EMT, “epithelial-mesenchymal transition” [47].

Our group particularly focused on the study of miR-205 involvement in breast cancer biology. Previous studies showed that miR-205 is significantly under-expressed in human breast cancer [34,48] and associated with the absence of vascular invasion [34], although it has also been shown to be up-regulated in other tumors types, such as ovarian cancer [49]. We recently demonstrated [40] that miR-205 is able to interfere with the HER receptor family-mediated survival pathway by directly targeting the HER3 receptor and thus inhibiting its downstream mediator Akt. In addition, other studies indicated that miR-205 is a negative regulator of the EMT, an early phase of the process of metastasis, targeting the transcription factors ZEB1 and ZEB2. Moreover, miR-205 also targets VEGF-A, a factor which plays a key role in the process of invasion and metastasis [48].

### 1.3. Triple Negative Breast Cancer

Breast cancer is a very heterogeneous disease. Perou *et al*., in 2000, classified breast cancer into five main categories, according to intrinsic gene signature: Luminal A, estrogen receptor (ER) and/or progesterone receptor (PR) positive, and human epidermal growth factor receptor 2 (HER2) negative; Luminal B, ER or PR positive and HER2 positive; HER2 overexpressing, basal-like and normal-like [50]. Further gene expression studies enriched the sub-classification of breast cancer [51] individuating additional entities such as the Claudin-low subtype, characterized by low expression of claudin proteins, proliferation genes and luminal markers, and high expression of ephitelial-to-mesenchymal transition markers and cancer stem-cell-like features [52]. Moreover gene expression profiling has also been used for the development of prognostic signatures such as Mammaprint and Oncotype DX [53–55], which are now enrolled in clinical trials.

However, a gap between achieved molecular knowledge and clinical applications still exists. Indeed, at the time of diagnosis, breast cancer classification and choice of adjuvant treatment still rely on traditional pathological parameters and immunoisthochemical (IHC) analysis of three main markers (ER, PR and HER2), which divide breast cancer into Luminal, HER2 positive, and Triple Negative Breast Cancer [56]. The latter is further stratified based on the expression of basal cytokeratins 5–6 and EGFR [57,58].

Triple Negative Breast Cancer (TNBC) is defined as ER-, PR- and HER2-negative. A first molecular characterization classified TNBCs as basal-like (50%), claudin-low (30%) and luminal A, B and HER2 subtypes for the remaining 20% [59]. Finally, Lehmann *et al*. in 2011 profiled more than 500 TNBCs classifying them in six different subtypes, according to intrinsic gene signature: basal 1 and 2, mesenchymal and mesenchymal stem cell-like, immunomodulatory and androgen pathway enriched [60]. TNBC accounts for 15%–20% of all breast cancers; it represents the most aggressive subtype with the most dire prognosis. Indeed, the pathological complete response (pCR) is achieved only in 20%–30% of cases after neoadjuvant chemotherapy and, among patients who do not achieve pCR, TNBC patients show poorer outcome compared to non-TNBC patients [61,62]. Moreover, TNBC patients have the highest percentage of early local relapse, especially between the first and third year after diagnosis [58]. Metastases are more aggressive compared to other subtypes and mainly occur in the viscera, in particular the lungs and brain [63], and to a lesser degree in bones [61].

Another major issue with TNBC is the lack of a specific target so that chemotherapy remains the standard of treatment; even if TNBC shows more chemosensitivity compared to ER-positive breast cancer, most of TNBC patients have residual disease in the breast and lymph nodes after neoadjuvant treatment. The absence of a targeted therapy renders these patients more susceptible to relapse and thus to poorer overall survival compared to other breast cancer subtypes.

Improvement in TNBC biology understanding, unraveled molecular pathways deregulated and with potential to be formed into drugs (Supplementary Figure S1). Gene amplification of *EGFR* and *VEGFA* and chromosomal deletion of *PTEN* and *RASA1* gene chromosomal region were described in [64,65], whereas the gene profile disclosed overexpression of *C-KIT* and interferon/immunoglobulin-related genes and low expression of *Bcl-2* [66], leading to the development of novel therapeutic approaches. However, the outcome of clinical trials conducted in the last years was controversial: the monoclonal anti-VEGF-A Bevacizumab, for example, appeared as one of the most promising novel agents; however, the promising effect in a neoadjuvant setting, in addiction to chemotherapy, in HER2-negative breast cancer patients [67] was not confirmed by a subsequent clinical study [68]. On the other hand, the encouraging results of the tyrosine kinase inhibitor Sunitinib, when used as single agent in a phase II clinical trial on metastatic breast cancer [69], resulted in totally disappointing results in a phase III clinical trial in a HER2-negative breast cancer population both as a single agent [70] or added to chemotherapy [71]. Unfortunately, also the monoclonal antibody against EGFR cetuximab and the TKI erlotinib only showed marginal effects [72,73].

These results could be in some part explained by differences in classification methods among different institutes and by a nonaccurate selection of patients; this means that patients enrolled in the clinical trials are not always the ones that could really benefit from the specific targeted therapy under investigation. Indeed, these speculations stress the importance of accuracy in patient selection and the need for robust molecular markers able to translate the knowledge coming from preclinical and *in silico* analyses into the clinics.

## 2. MicroRNAs and TNBC

### 2.1. Profiling Studies

Starting from the first profile that identified microRNAs deregulated in breast cancer specimens compared to normal tissues [34], numerous profiles have been performed on tissue samples in an attempt to individuate microRNA signature with clinical value. However, results are often controversial, leaving the question open as to whether microRNA profiling is reliable or not for breast cancer stratification.

Signatures of differentially expressed microRNAs were found between basal and luminal breast cancer subtypes [74,75], and could specifically classify estrogen receptor (ER), progesterone receptor (PR) and HER2/neu receptor status [34,76,77].

In a more recent retrospective analysis on lymph node negative breast cancer tissues, hierarchical clustering based on microRNA profiling was able to identify four main subgroups. However, the TNBC was revealed as the most prominent with a unique microRNA signature. The highest overall classification values by analysis of variance followed by cross-validation was indeed found for cytokeratin 5 and 6, triple negative and estrogen receptor [78].

Farazi *et al*. in 2011 confirmed in a set of normal, DCIS and invasive breast cancer specimens, the tendency of TNBCs to cluster, according to microRNA profile, as a distinct group with respect to tumor samples found to be positive to one or more IHC marker. However, the strength of this microRNA profiling in breast cancer sub-classification did not compare well with mRNA profiling [79].

Further analysis of Farazi’s dataset by Croce’s group [80] revealed modulation of nine microRNAs differentiating invasive from *in situ* carcinoma, including miR-210, confirmed activation of miR-17-92 family in IDC TNBC and further identified additional microRNAs differentially expressed in this subtype, such as upregulation of miR-200c. Interestingly, also overexpressed microRNAs have been identified in TNBC, even though the highly undifferentiated nature of this tumor subgroup would suggest a global downregulation of microRNAs [81] (Dvinge *et al*., Nature 2013).

A more recent study attempted to individuate microRNAs associated to hypoxia and angiogenesis by evaluating microRNA heterogeneously expressed within a tumor, since they could reflect a different microvessel distribution and thus different hypoxic conditions within the tumor itself. They found sets of microRNAs heterogeneously expressed from the edge to the center of the tumor for each clinical-pathological subtype, with a set of 50 microRNAs for TNBC. Among these, miR-20a and miR-20b appeared the most interesting, as they are predicted to target VEGF-A and HIF-1a [82].

The first study totally focused on TNBC with the most consistent results came from a large-scale profile of Cascione *et al*. in 2013 [83]. In a first set of primary TNBC and normal tissues, the microRNA profile revealed 116 deregulated microRNAs, among which miR-106b, the cluster miR-17/92, miR-200 family (miR-200a, miR-200b and miR-200c), miR-21 and miR-155 were the most up-modulated while let-7b, let-7c, miR-126, miR-145 and miR-205 were the most down-modulated [83]. A second set of TNBC-associated lymph node metastasis and normal tissues allowed instead the identification of a set of six miRNAs differentially expressed in the metastatic tissues (miR-424, miR-125a-5P, miR-627, miR-579, let-7g, miR-101), with miR-424 and miR-125a differentially expressed only in the metastasis *vs*. primary tumor and in normal tissue *vs*. metastasis, respectively [83].

Interestingly, some of the miRNAs that associate with the luminal or basal subtype reflect their epithelial and myoepithelial origins, respectively. For instance, the miR-200 family associates with the luminal subtype. This is not surprising since they directly target the EMT regulators ZEB1 and ZEB2 [47], whereas miR-145 and miR-205, preferentially expressed in normal myoepithelial cells, are dramatically reduced in basal-like triple negative tumors (ER-/PR-/HER2-), suggesting that this expression change might be a consequence of disease progression in this subtype [75].

Among the putative mechanisms regulating microRNA expression, abnormalities in the biogenesis machinery have been reported. However, the currently available data about TNBC are both few and controversial: indeed, whereas Dedes K.J. *et al*. [84] report Dicer mRNA downregolation, consistent with the hypothesis of an oncosuppressive role of the vast majority of microRNAs, Passon N. *et al*. [85] detected high IHC staining of both Drosha and Dicer. Interestingly, Lin28B, being able to selectively inhibit let-7 processing by Dicer and function, is specifically expressed in TNBC [86], acting as an oncogene. Even microRNAs themselves can control the biogenesis machinery, as the oncogenic miR-103/107 is able to directly target Dicer [87].

The use of *in silico* bioinformatic prediction tools and integrated microRNA:mRNA computational analysis generated complex networks with the attempt to collocate the microRNAs individuated by profiling studies in cancer-related pathways [81,88]. The potential of these networks to unravel new targets and/or therapeutical approaches is promising but at the same time still far off; indeed, only a small number of all these microRNAs are supported by functional or clinical evidence in TNBC.

### 2.2. MiR and TNBC—Functional Evidence

For most of the microRNAs that come from profiling studies, only *in silico* analysis has been reported that describes predictive interactions with the main biological processes related to TNBC biology. However, there is a limited promising number of microRNAs supported by validated experimental data (Table 1, Supplementary Figure S2).

Among the tumor suppressor microRNAs, the miR-200 family, miR-205 and the let-7 family are the most described.

The miR-200 family appears to be one of the most interesting players in TNBC biology; it was previously described as up-modulated in breast cancer where its over-expression was correlated with lymph node positivity and metastasis [102]. Among the different breast cancer subtypes, the miR-200 family was shown to be down-modulated in metaplastic carcinoma [103], one the most mesenchymal and undifferentiated breast cancers characterized by TNBC phenotype. However, functional *in vitro* evidence that describes the miR-200 family as a tumor suppressor in TNBC, in concomitant with the very low expression levels in the Basal B breast cancer cell lines [104], *in vitro* model of metaplastic, claudin-low and mesenchymal breast tumors [60], seems to be in contrast with its overexpression in tumor samples [88]. This highlights, therefore, the still controversial role of miR-200 family in breast cancer. Indeed, in concomitant with miR-205, the miR-200 family is a well known negative regulator of the epithelial-mesenchymal transition (EMT), through the direct targeting of Zeb1/Zeb2, two of the key trascriptional factors that guide the process [47]. The MiR-200 family is also down-modulated in stem-cells and breast cancer stem cells [105] and has been demonstrated necessary for the mammary cell differentation process [89]; in particular, downmodulation of miR-200a and miR-200b is observed in a developmental stage of mammary epithelial cell lines which shows correlation in gene expression with basal-like and poor prognosis breast cancer [106]. Furthermore, the miR-200 family, in particular miR-200c, inhibits cancer cell migration [107], invasion [108] and reverts the anoikisis resistance [90,91] that is frequently observed in aggressive carcinoma cells where it correlates with EMT [109,110]. Finally, low expression levels of miR-200c associated with poor response to chemotherapy [111] and radiotherapy [112].

The miR-205 exerts, instead, a clearer tumor-suppressive role. Our group described its down-modulation in TNBC, in particular in the claudin-low subgroup, to be in agreement with other studies [113]. We demonstrated that the ectopic expression of miR-205 reduces proliferation, cell cycle progression and clonogenic potential *in vitro*, and inhibits tumor growth *in vivo*, partially by targeting of E2F1, master regulator of cell cycle progression, and LAMC1, the component of extracellular matrix involved in cell adhesion, proliferation and migration. Finally, we demonstrated that the transcription of miR-205 is directly induced by p53 [92].

MiR-203, which has been down regulated in several cancers [114–117], also presents low levels in TNBC cell lines. Ectopic expression reduces cell proliferation and migration by targeting BIRC5, which is frequently up-regulated and is associated with poor clinical outcome in breast cancer [118,119], and LASP1, and found to be over-expressed in metastatic breast cancer [120], respectively [93].

The Let-7 family is one of the best known microRNA in the cancer field. It regulates numerous oncogenes, like RAS, MYC and HMGA2 [121], it was found to be down-modulated in different cancer types [122] and restoration in breast cancer inhibited tumor growth [123], validating its role as tumor-suppressor. Moreover, up-modulation of let-7 and miR-200 families was demonstrated to partially mediate the action of garcinol [124], a natural chemical compound with demonstrated anticancer activity [125], in *in vivo* and *in vitro* TNBC models. To support the importance of let-7 family in TNBC, recently the *KRAS*-variant, a germline single nucleotide polymorphism (SNP) mutation in a let-7 complementary site in the 3′UTR of KRAS [126,127], was associated with risk in developing TNBC [128]. Finally, LIN28B, regulator of let-7 biogenesis by blocking the pri-let-7 processing in the nucleus [86], was associated with advanced disease with aggressive and poorly differentiated phenotypes [129–131].

Besides the afore-mentioned microRNAs, it is also worth mentioning miR-31, miR-146, miR-146b and miR-34a. MiR-31 plays a specific metastatic suppressor role through the regulation of pro-metastatic oncogenes like WAVE3 [94], RhoA, Radexin [95]. Its expression in breast cancer decreases from early stage to aggressive phenotypes [94] becoming undetectable in metastatic breast cancer. The down-modulation in TNBC cell lines, with particular attention to basal subtype, was partially attributed to epigenetical hypermethylation of LOC554202, the miR-31 host gene [132]. In contrast, in the mouse model, restoration of miR-31 inhibits metastatic potential of TNBC cell lines without affecting primary tumor growth [95]. In addition to its role in the metastastic process, miR-31 overexpression also induces apoptosis and increases chemo- and radiosensitivity in TNBC cell lines by direct inhibition of PRKCE, a positive regulator of Bcl-2 [133]. Inverse correlation between miR-31 and Bcl-2 was found by exploiting a public dataset of breast cancer patients [96]. Finally, miR-34a, one of the most down-modulated by a microRNA profiling study between luminal and Basal-B cell lines, resulted particularly under-expressed in the TNBC cell line where it inversely correlates with AXL and mediates impairment in AXL-mediated migration [97].

Moving to oncomiRs, the well-established up-modulated miR-21 and miR-155 still play a marginal role in TNBC. MiR-21, was found to be over-expressed in different cancer types, including breast cancer [34,35,134]. It does not differ according to receptoral status [135], and only in TNBC was a trend between high miR-21 level and poor prognosis described [113]. Regarding miR-155, in breast tumors it induces angiogenesis and tumor growth and its up-regulation is associated with metastasis, late-stage/high grade tumor and poor prognosis [136]. Interestingly, a significant proportion of TNBC show high expression of miR-155 (moreover, it was shown to be epigenetically regulated by BRCA1 and up-regulated in BRCA1-deficient or BRCA-mutant breast cancer [137]) and stimulated by pro-oncogenic stimuli such as hypoxia, both risk factors for TNBC. Even if the exact role of miR-155 in TNBC is still poorly elucidated, this evidence suggests a deeper investigation of its the putative role in TNBC, both as marker and therapeutic target.

Recent data described miR-181a, which is overexpressed in TNBC [98], as metastamiR. It belongs to the miR-181 family, which also comprises miR-181b, -c and -d; all the family members are positively regulated by TGF-β, even if the exact mechanism by which TGF-β regulates each microRNA is not completely understood. Down-modulation of miR-181a inhibits TGF-β mediated EMT, invasion and migration, also reverting anoikisis resistance in breast cancer cells; high levels were then associated with shorter disease-free survival of breast cancer patients, who tested negative for ErbB2 amplification [98]. Moreover miR-181a/b expression sensitizes to cisplatin [138] and to PARP inhibitors [99] by dampening the DNA double-strand-breaks repair, thus representing a possible marker of BRCAness. Quantification of miR-181a levels could then be useful for determining basal-like and TNBC patients’ sensitivity to platinum-derived compounds and who could really benefit from PARP inhibitors treatments.

miR-146 and miR-146b-5p were validated as regulator of BRCA-1 and found up-modulated in TN and basal-like breast cancer cell lines, being responsible for BRCA1-mediated effects on proliferation and homologous recombination [100]. Finally, miR-182 was found up-modulated in TNBC tissues and cell lines, antagonism inhibited cell proliferation and invasion, and induced apoptosis [101], confirming its oncogenic role previously described in other tumor types [139,140].

### 2.3. MiR and TNBC—Prognostic Value

MicroRNAs have already demonstrated their reliability as biomarkers for prognosis in response to therapy or therapeutic tools in other cancer types. In lung cancer, low levels of let-7a and the high levels of miR-155 are indicative of worse prognosis [141] while a robust 7-miRNA signature can predict overall survival (OS) and relapse-free survival in gastric cancer [142]. In melanomas, low miR-191 and high miR-193a levels were associated with a significantly shorter survival time [143]. In contrast, high levels of miR-21, in addition to being an indicator of poor prognosis in several cancers [115,144,145], is sufficient to predict poor response to adjuvant chemotherapy in adenocarcinoma [115].

With the improvement in stability, specificity and biodistribution of microRNAs in *in vivo* systems [146–148], the use of microRNAs in therapy became more pragmatic. In particular, the miR-34a represents the most suitable therapeutic target [149]. Systemic delivery of miR-34a through liposomal-like vehicle in mouse models led to a huge impact on tumor growth in lung cancer [150] and total regression in hepatocellular carcinoma without side effects. Mirna Therapeutics has already announced the arrival of MRX34, the liposomal-formulated miR-34a mimics used for the study related to HCC, in a Phase I clinical trial.

A recent report by Caldas group [81] describes an integrated analysis of microRNA and mRNA expression with genomic, epigenetic and clinical features in a large set of samples. They showed that, in breast cancer, single microRNAs seem to be poor prognostic indicators, and specific signatures can be identified, but they seem consistently prognostic only in the subgroup of breast cancer devoid of copy number alteration (CNAs), the so called iClust4, as identified in the Metabric cohort [151]. Moreover, this signature mainly includes novel microRNAs, which may reflect their discovery by sequencing high grade tumors.

However, a number of known microRNAs have been identified as prognostic in breast cancer, including the Triple Negative subgroup, where a set of four microRNAs were found to associate with OS. In detail, up-regulation of miR-16, miR-155 or miR-374 correlated with better prognosis while down-modulation of miR-125b correlated with worse prognosis. Furthermore, a set of seven microRNAs were associated with distan disease free survival (DDFS). Cascione *et al*. identified three “risk-associated” miRs (miR-125b, miR-655 and miR-421) and four “protective” miRNAs (miR-16, miR-374a, miR-374b and miR-497) which negatively and positively correlated with DDFS, respectively [83]. However, these signatures require further validation in a second set on TNBC samples.

MiR-210, over-expressed in TNBC compared to ER+ [113], was described as an independent prognostic factor in TNBC; low levels of miR-210 showed better distant-free-survival (DFS) in TNBC and no relapse in the following 5 years after surgery in node negative TNBC [152]. MiR-34b, a member of the p53-regulated miR-34 family [153], is negatively associated with DFS and OS [154]. However this observation is not consistent with previously reported tumor-suppressor roles of the miR-34 family in cancer. Interestingly, there is a positive correlation of miR-25, miR-10b, miR-130b, miR-1274a, and miR-Plus-1030 and a negative correlation of miR-29c, which is downregulated in ER-/cytokeratin 5 and 6 positive tumors, with proliferation in a set of lymph node negative breast cancer [78], since proliferation represents the strongest single prognostic indicator in this group of patients [155].

## 3. Conclusions

The molecular and phenotypical heterogeneity that characterizes Triple Negative Breast Cancer has been partially clarified, and the complexity of signal networks driving the biology of a tumor subgroup for which the absence of targets for a specific therapy certainly contributes to a poor clinical outcome has been discussed.

In this scenario, microRNAs might represent not only an additional level of complexity in the molecular portrait of TNBC, contributing to tumor comprehension and subclassification, but, more importantly, they might represent easily detectable biomarkers to predict prognosis and response to therapy. Certainly, their potential as biomarkers needs to be validated in different cohorts of samples, taking into account the evidence that profiling signatures are probably more statistically significant that single miRNAs in predicting outcome.

Moreover, even though the available, up to date data are almost exclusively pre-clinical evidence, the application of miRNAs in therapy as adjuvant tools of targets seems convincing and promising.

## Supplementary Information



## Figures and Tables

**Table 1:** Summary of microRNAs involved in Triple Negative Breast Cancer (TNBC), with relative validated targets and biological functions.

MicroRNA	Validated target(s)	Main biological function(s) in TNBC	Reference
**Tumor suppressor**			
miR-200a/b	Zeb1/Zeb2, Suz 12, EphA2	Stimulation of differentiation in undifferentiated mammary epithelial cell line	[89]
miR-200c	Zeb1/Zeb2	Inhibition of EMT	[47]
	MSN; FN1	Suppression of migration	[90]
	TrkB	Reversion of anoikisis resistance	[90,91]
miR-205	E2F1; LAMC1	Reduction of proliferation, cell cycle and tumor growth	[92]
miR-203	BIRC5	Reduction of proliferation	[93]
	LASP1	Inhibition of migration	
miR-31	WAVE3; RhoA; Radexin	Reduction of metastatic potential	[94,95]
	PRKCE	Induction of apoptosis and enhancement of chemo- and radiosensitivity	[96]
miR-34a	AXL	Impairment of migration	[97]

**OncomiR**			
miR-181a/b	Bim	Inhibition of anoikisis	[98]
	ATM	Impairment of DNA double-strand-breaks repair	[99]
MiR-146 and miR-146b-5p	BRCA1	control of BRCA1-mediated proliferation and homologous recombination	[100]
miR-182	PFN1	Inhibition of cell proliferation and invasion	[101]
		Induction of apoptosis	
