# Supplementary material for: MicroRNAs and Triple Negative Breast Cancer
Source: Int J Mol Sci. 2013 Nov 11;14(11):22202–20. doi: 10.3390/ijms141122202 (PMC3856060; doi:10.3390/ijms141122202)
Supplement: Supplementary file 1 [file ijms-14-22202-s001.pdf]

Supplementary Information

Figure S1. Novel therapeutic strategies in TNBC treatment. Schematic representation of some of the most important deregulated pathways in TNBC, due to genomic amplification (EGFR, VEGFA), chromosomal deletion (PTEN) or gene overexpression (c-KIT). The key regulatory molecules (highlight in **red**) of these unravelled pathways have been recently testing as therapeutic targets in several clinical trials.

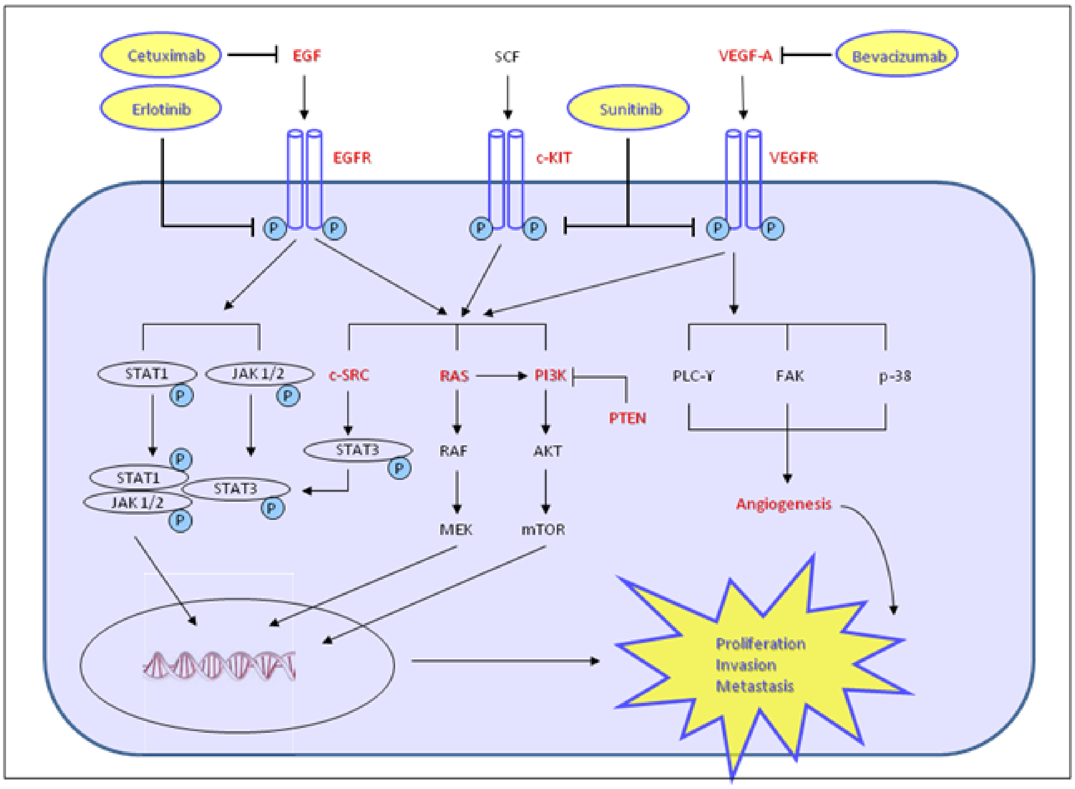

Figure S2. MicroRNAs deregulated in TNBC and their impact on cell response and behaviour. (**A**) Genotoxic or anoikis stimuli induce in a normal cell the arrest of cell cycle and eventually activate programmed cell death (lines in **blue** and **red**). Moreover stimulation of receptors that trigger activation of the locomotory apparatus, among which actin remodelling and myosin fibers formation, shifts the cell toward a migratory phenotype (lines in **orange**); (**B**) Stable alterations in microRNA expression, as occurs in cancer cells, direct cell fate toward an uncontrolled proliferation by ignoring apoptosis/anoikis stimuli (lines in **blue** and **red**). In alternative the down-modulation of microRNAs regulating molecules involved in migration and locomotion confers an aggressive phenotype more suitable for invasion and metastasis (lines in **orange**).

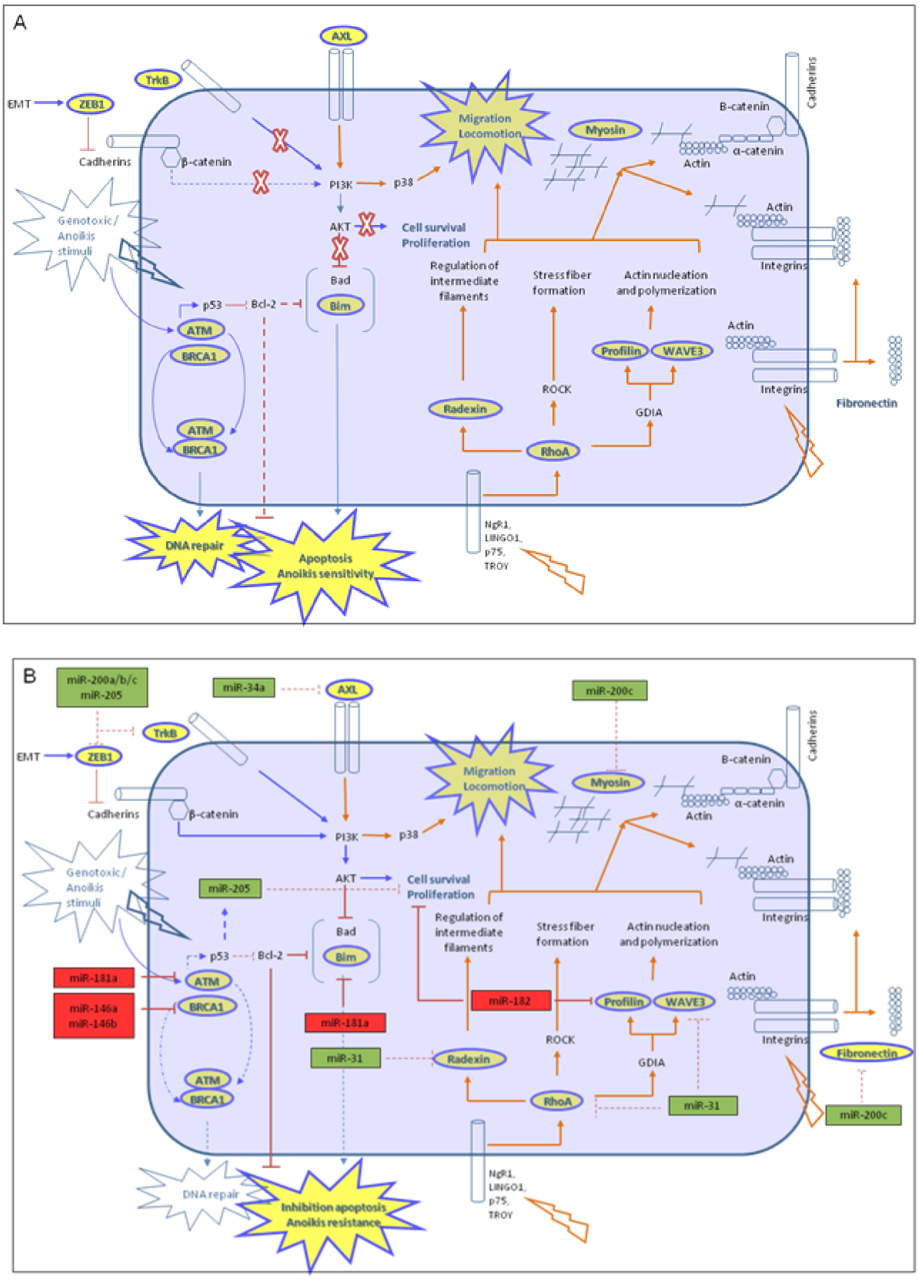

© 2013 by the authors; licensee MDPI, Basel, Switzerland. This article is an open access article distributed under the terms and conditions of the Creative Commons Attribution license (http://creativecommons.org/licenses/by/3.0/).
